# Context-dependent antioxidant defense system (ADS)-based stress memory in response to recurrent environmental challenges in congeneric invasive species

**DOI:** 10.1007/s42995-024-00228-y

**Published:** 2024-05-08

**Authors:** Hanxi Li, Xuena Huang, Aibin Zhan

**Affiliations:** 1 Research Center for Eco-Environmental Sciences, Chinese Academy of Sciences, Beijing, 100085 China; 2 University of Chinese Academy of Sciences, Beijing, 100049 China

**Keywords:** Antioxidant defense, Ascidian, Biological invasion, Environmental stress, Stress memory

## Abstract

Marine ecosystems are facing escalating environmental fluctuations owing to climate change and human activities, imposing pressures on marine species. To withstand recurring environmental challenges, marine organisms, especially benthic species lacking behavioral choices to select optimal habitats, have to utilize well-established strategies such as the antioxidant defense system (ADS) to ensure their survival. Therefore, understanding of the mechanisms governing the ADS-based response is essential for gaining insights into adaptive strategies for managing environmental challenges. Here we conducted a comparative analysis of the physiological and transcriptional responses based on the ADS during two rounds of 'hypersalinity-recovery' challenges in two model congeneric invasive ascidians, *Ciona robusta* and *C. savignyi*. Our results demonstrated that *C. savignyi* exhibited higher tolerance and resistance to salinity stresses at the physiological level, while *C. robusta* demonstrated heightened responses at the transcriptional level. We observed distinct transcriptional responses, particularly in the utilization of two superoxide dismutase (SOD) isoforms. Both *Ciona* species developed physiological stress memory with elevated total SOD (T-SOD) and glutathione (GSH) responses, while only *C. robusta* demonstrated transcriptional stress memory. The regulatory distinctions within the Nrf2-Keap1 signalling pathway likely explain the formation disparity of transcriptional stress memory between both *Ciona* species. These findings support the 'context-dependent stress memory hypothesis', emphasizing the emergence of species-specific stress memory at diverse regulatory levels in response to recurrent environmental challenges. Our results enhance our understanding of the mechanisms of environmental challenge management in marine species, particularly those related to the ADS.

**Supplementary Information:**

The online version contains supplementary material available at 10.1007/s42995-024-00228-y.

## Introduction

Owing to global climate change and human activities, the increasing frequency and intensity of environmental changes pose an unprecedented threat to marine species, particularly sessile ones lacking behavioral choices to select optimal habitats (Hackerott et al. 2021; Hughes et al. 2018; Pickering et al. 2013). In response to these environmental challenges, marine sessile organisms have to rapidly implement available strategies to mitigate the adverse effects of environmental stressors (Blewett et al. 2022; Snell-Rood et al. 2018; Somero 2022). Often, acute environmental challenges induce the redox imbalance and excessive accumulation of reactive oxygen species (ROS), leading to oxidative stresses and potential damages to organisms (Hofmann and Todgham 2010; Schieber and Chandel 2014; Sies 2019). To counteract the excess ROS, organisms have developed an efficient antioxidant defense system (ADS) as their primary line of defense, enabling them to regulate the imbalanced redox state and maintain homeostasis (Lukić et al. 2020; Martin and Nia 2016). Considering the intricate evolutionary patterns of physiological antioxidant defense mechanisms, which have substantially contributed to the development of defense strategies against a wide array of biotic and abiotic stressors, it becomes evident that the antioxidant capacity plays a pivotal role in determining an organism's environmental tolerance (Portner and Farrell 2008; Somero 2012). Consequently, deciphering the intricate mechanisms governing the ADS-based response to environmental perturbations holds promise in providing insights into adaptive strategies for managing environmental challenges (Carlson et al. 2014; Nandamuri et al. 2017).

The ADS comprises a sophisticated interactive network of enzymatic defenses and low-molecular-weight scavengers (Moreira et al. 2016; Sies et al. 2017; Zheng et al. 2016). The key antioxidant enzymes consist of superoxide dismutase (SOD), which is responsible for scavenging superoxide anions; catalase (CAT), which facilitates the breakdown of H_2_O_2_; and glutathione peroxidase (GPx), which is effective against both H_2_O_2_ and organic peroxides (Davies 2016; Sthijns et al. 2016). Additionally, non-enzymatic antioxidant scavengers such as glutathione (GSH), ascorbate, α-tocopherol, and carotenoids also play crucial roles by directly or indirectly participating in the enzymatic reactions of antioxidant enzymes in ROS neutralization. For example, the antioxidative enzyme glutathione S-transferase (GST) plays a role in detoxifying reactive oxygen species by facilitating the conjugation of glutathione (GSH) with various electrophilic substrates (Lesser 2006; Sies et al. 2017; Storey 1996). For instance, organisms exposed to salinity stresses have to increase their bioenergetic expenditure, including aerobic metabolism and respiration, to regulate ion and water flux across biological membranes. This adjustment entails the excessive load of ROS, triggering the activation of antioxidant enzymes such as SOD and CAT and an elevation in GSH concentration (Detree and Gallardo-Escarate 2018; Martin and Nia 2016; Rivera-Ingraham and Lignot 2017). Through the cellular ADS-based response, which involves boosting the catalytic activity of antioxidative enzymes or the de novo synthesis of non-enzymatic antioxidants, organisms under stresses can rapidly (often within minutes) mitigate the oxygen toxicity and maintain the redox potential (Shadel and Horvath 2015; Sthijns et al. 2016).

In addition to changes in cellular physiology driven by the activation of antioxidant enzyme activity, the response of organisms also involves coordinated alterations in the expression patterns of antioxidant-related genes (Ding et al. 2012). The transcriptional regulation of many antioxidant-related genes, such as SOD, CAT, GPx, and GST, is orchestrated through the ROS-activated transcription factors, particularly Nrf2 [Nuclear factor (erythroid-derived 2)-like 2, also known as NFE2L2] via the Nrf2-Keap1 (Kelch-like-ECH-associated protein 1) signaling pathway (Battino et al. 2018; Davies 2016; Ma 2013; Ristow 2014). For example, transcriptome sequencing studies in crabs exposed to hypersalinity revealed that oxidative stress-related pathways, particularly the Nrf2-mediated oxidative stress response, were enriched in gills (Li et al. 2014). Another study conducted on the large yellow croaker *Pseudosciaena crocea* demonstrated the engagement of zinc-induced antioxidant defences, manifesting changes at both enzymatic and transcriptional levels, as well as the transcriptional regulation of the Nrf2-Keap1 signalling pathway (Zheng et al. 2016). Collectively, the intricate regulation of the ADS, involving both cellular physiological and transcriptional mechanisms, empowers organisms to mitigate imbalanced redox states, counter continuous abiotic changes, and ultimately maintain adaptive homeostasis (Ma 2013; Shadel and Horvath 2015). However, many studies have focused solely on one aspect of antioxidant responses, often either mRNA expression or enzymatic activity. There is now a need to integrate responses across multiple layers of the antioxidant defense to thoroughly evaluate their effectiveness in defending against environmental stressors.

As the frequency of recurrent environmental stresses continues to rise, a growing body of research suggests that organisms have the capacity to establish alternative responses based on their exposure history (Crisp et al. 2016; da Silva et al. 2019; Oberkofler et al. 2021; Pfennig 2021). This phenomenon, known as 'stress memory', has been observed in a variety of species across different taxa (Crisp et al. 2016; Ho et al. 2020; Oberkofler et al. 2021; Wang et al. 2022). Furthermore, plastic responses based on the ADS also exhibit stress memory for recurrent environmental challenges (Liu et al. 2022). For example, prior exposure to drought conditions enhanced the activities of antioxidative enzymes in *Alopecurus pratensis* during subsequent drought periods, thus improving its drought tolerance (Lukić et al. 2020). In the case of the mussel *Mytilus galloprovinciali*, heat hardening enhanced the mitochondrial respiration potential and oxidative defense capacity in the mantle of thermally stressed individuals (Georgoulis et al. 2021). Likewise, differential gene expression responses of GPx were observed in individuals exposed to microplastics for the first time compared to those repeatedly exposed, suggesting the establishment of transcriptional stress memory within the ADS following repeated microplastic exposure (Detree and Gallardo-Escarate 2018). Nevertheless, it remains to be determined whether the development of stress memory is species- or challenge-specific and whether it occurs at various regulatory levels, such as physiological and transcriptional responses, when organisms confront environmental challenges.

Invasive ascidians, such as the genus *Ciona*, have become model species for studying environmental response mechanisms during biological invasions (Zhan et al. 2015). *Ciona robusta* and *C. savignyi*, thought to originate from the Northwest Pacific Ocean, have successfully colonized a wide range of coasts with varied environmental conditions globally (Fofonoff et al. 2018; Zhan et al. 2015). Additionally, *C. robusta*, in particular, exhibits a wider geographical distribution, with confirmed occurrences in the Mediterranean Sea, South Africa, and even the Red Sea which is characterized by its high temperature of > 27 °C and high salinity of 41 (Berna et al. 2009; Shenkar et al. 2018). The successful colonization in the Red Sea highlights the remarkable tolerance of *C. robusta* to harsh environments, particularly hypersalinity (Chen et al. 2018; Shenkar et al. 2018). Furthermore, for marine invasive species transported through shipping, salinity changes in ballast tanks can be both transient and frequent, with fluctuations of up to 15 occurring within a few days of the voyage (Briski et al. 2013; Lin et al. 2020). *Ciona* species demonstrate remarkable resilience in surviving these challenging environments with stressors that vary widely and change rapidly during invasions, highlighting their adaptive capabilities when confronted with environmental challenges (Zhan et al. 2015). The remarkable adaptative capacities, along with the similarities and distinctions between *C. robusta* and *C. savignyi* during their invasions, make them excellent comparative models for studying stress memory based on the ADS in response to environmental challenges.

Using the two invasive model ascidians, *C. robusta* and *C. savignyi*, here we conducted two rounds of 'hypersalinity-recovery' experiments to simulate repeated environmental changes encountered during the invasion process. By integrating both physiological and transcriptional responses of the ADS, we aim to test whether the development of stress memory is species-specific at diverse regulatory levels (i.e., context-dependent stress memory hypothesis). Specifically, we (1) investigated the profiles of antioxidative physiological and transcriptional responses and associated regulatory mechanisms, and (2) explored the distinct response patterns between two rounds of stresses and between the two *Ciona* species. Our results in this study are expected to provide insights into the maintenance of homeostasis in response to environmental challenges and the mechanisms of phenotypic plasticity responsible for invasion success.

## Materials and methods

### Sample collection and acclimation

Adult *Ciona robusta and C. savignyi* were collected from scallop cages in the Longwangtang maricultural area of Dalian, Liaoning province, China (38°48′53″ N, 121°24′06″ E) in September 2018, and then immediately transferred to the laboratory. Since these two species co-occurred on the substrates, we initially separated them based on their morphological characteristics (Bhattachan et al. 2020) and then confirmed the identification using molecular methods (Smith et al. 2012; Zhan et al. 2010). The ascidians were acclimated in ambient seawater at a temperature of 20.2 ± 0.8 °C and a salinity of 29.8 ± 0.6 for 1 week. Throughout the experiment, the ascidians were fed with a mixture of powdered, wall-broken Chlorella (*Chlorella* spp.) and spirulina (*Arthrospira platensis*) twice daily.

**Fig. 1:**
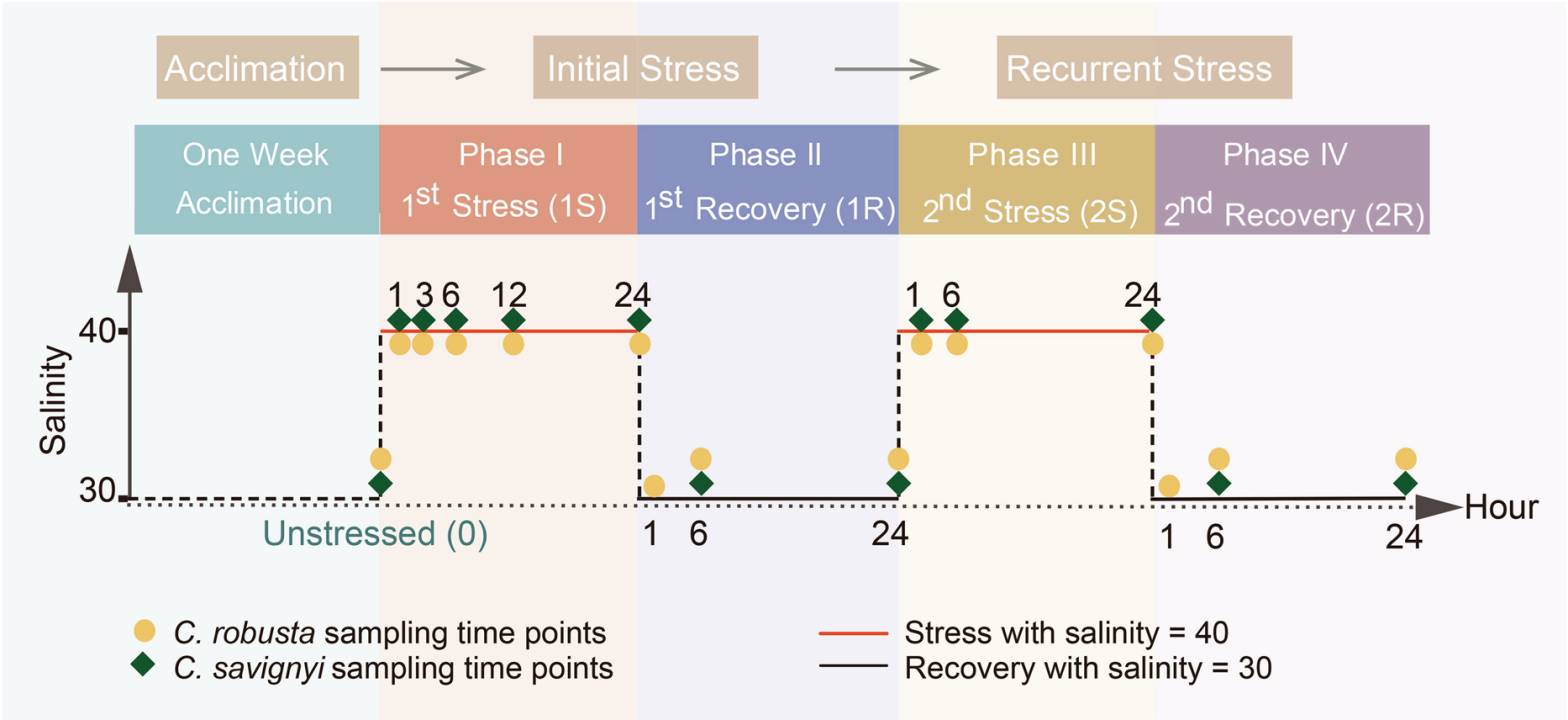
Schematic representation of the experimental design. Both *Ciona* species were placed in two equivoluminal automatic constant temperature incubators after 1-week acclimation with the setting of 40 as the initial hyper-salinity stress (1S). After 24 h stress, the salinity of the incubators was set back to 30 with an additional 24 h as the recovery period (1R). This stress-recovery cycling was then repeated for another time in the same experimental groups of ascidians. The yellow and green points indicate the sampling time of *C. robusta* and *C. savignyi,* respectively

To avoid harming ascidians during tissue sampling for molecular identification, all individuals subjected to salinity challenges were initially identified based on morphology. After the stress exposure and subsequent molecular identification, most of the samples were accurately identified using both morphological and molecular methods. However, four *C. robusta* specimens (from 1R-1 and 2R-1) were erroneously classified as *C. savignyi* based on their morphological characteristics. Consequently, these four individuals were excluded from further analyses (Fig. 1).

### Two rounds of ‘hypersaline stress-recovery’ treatment

Hypersaline stress was set at 40, which corresponds to the salinity tolerance of *C. robusta* and is consistent with conditions in the Red Sea, where recent invasions by *C. robusta* have occurred (Chen et al. 2018; Shenkar et al. 2018). Seawater with a salinity of 40 was prepared by adding sea salt (Blue Grand Star, Jiangxi Haiding Technology Co., Ltd.) into natural seawater and was monitored by a salinometer. In our stress treatment experiments, we conducted separate but simultaneous trials with the two ascidian species. After acclimating for a week, we randomly selected six ascidians of each species to form the unstressed groups, while the remaining ascidians were transferred from natural seawater to 40 seawater and maintained for 24 h (1S phase). Subsequently, the ascidians in 40 seawater were returned to natural seawater at 30 salinity for another 24 h (1R phase). This process was followed by a second identical cycle of hypersaline stress (2S phase) and recovery (2R phase). During each 24-h phase, we collected six individuals from both species at three time points: 1, 6, and 24 h. Additionally, two more sampling points were set at 3 h and 12 h after the first stress (Fig. 1). All collected ascidian individuals were dissected on sterile culture dishes, with the pharynx muscle divided into two parts for RNA isolation and physiological indicator measurement. All collected samples were immediately frozen in liquid nitrogen and then stored at – 80 °C.

### Antioxidant indicators assessment

To prepare tissue supernatants for antioxidant indicator analysis, ~ 80 mg of pharynx muscle of each sample was homogenized in 0.9% normal saline solution and centrifuged at 2500 r/min for 10 min. Then, the supernatants were immediately used for assessing total protein (TP) amount, malondialdehyde (MDA) concentration, total antioxidative capacity (T-AOC), total superoxide dismutase activity (T-SOD), catalase (CAT) activity, and glutathione (GSH) concentration, using an assay kit (A045-2, A042-1, A003-1, A001-1, A007-2 and A006-1; Nanjing Jiancheng Bioengineering Institute) according to the instructions. TP concentration was determined using the Coomassie brilliant blue staining method, while T-SOD was measured with the xanthine oxidase method, CAT using the ultraviolet method, and GSH and MDA with the dithiol dinitrobenzoic acid method and thiobarbituric acid method, respectively. All these indicators were subsequently measured using an ultraviolet spectrophotometer (DR6000, HACH Inc., USA).

### RNA extraction and gene expression of antioxidant-related genes

Total RNA was extracted from 50 to 100 mg pharynx muscle by TRIzol reagent (Ambion, USA) according to the standard protocol. RNA integrity and concentration were determined by agarose gel electrophoresis and Nano 2000 spectrophotometer, respectively (Nanodrop Technologies, USA). Subsequently, we reverse-transcribed 3 μg of total RNA into cDNA using the PrimerScriptII cDNA Synthesis Kit (Takara, Japan). MnSOD, Cu/ZnSOD, CAT, GpX, and GST were selected as candidate ADS-related genes due to their established importance in their response to oxidative stress. Additionally, the transcription factors Nrf2 and Keap1 of the Nrf2-Keap1 signaling pathway were also selected. All seven genes served as relevant markers for investigating the underlying molecular mechanisms associated with the antioxidant stress response in this study. The *β*-actin gene of *C. robusta* (Fujikawa et al. 2010) and RPL17, 60S ribosomal subunit gene of *C. savignyi* (Huang et al. 2016) were selected as the internal reference gene for normalization. The mRNA sequences of all genes were downloaded from Ensembl (http://asia.ensembl.org/index.html). The gene-specific primers were designed using Primer Premier 5.0. The amplification efficiencies of all primer pairs were checked by the standard curve method (Supplementary Table S1).

Quantitative Real-time PCR (qRCR) was executed with FastStart Essential DNA Green Master (Roche Applied Science, Germany) on Roche LightCycler® 96 detection system (Roche Applied Science, Germany). The qPCR reaction consisted of 20 μL, comprising 10 μL SYBR Green Master Mix, 8 μL PCR-free water, 0.5 μL of each forward and reverse primer, and 1 μL cDNA template. The thermocycling condition consisted of 95 °C for 2 min, followed by 40 cycles of 95 °C for 10 s; 60 °C for 10 s; 72 °C for 15 s, and a final extension at 72 °C for 5 min. We used an equal quantity of RNA from biological replicates at each time point for qPCR DNA templates, and we conducted three technical replicates for each cDNA template. Additionally, we established negative controls with no template to ensure that no exogenous nucleic acid contamination was introduced during the experiment.

We also calculated the Pearson correlation coefficients between each physiological and transcriptional ADS-related indicator for both *Ciona* species. Additionally, we performed further transcription factor binding site prediction and Protein–Protein Interaction (PPI) network analysis. The antioxidant response elements (AREs), as transcription factor binding sites of Nrf2 in the Nrf2-Keap1 signal pathway, were predicted within 2000 bp upstream of the transcriptional start site of each gene by MatInspector tool in Genomatix Software Suite (http://www.genomatix.de), and the number and position of AREs were obtained. Additionally, we constructed a protein–protein interaction (PPI) network to help identify the potential regulatory relationship among genes in the Nrf2-keap1 signaling pathway by online program STRING v.12.0 with the confidence score > 0.15 (Szklarczyk et al. 2019), and further visualized by Cytoscape (v. 3.9.1).

### DNA substitution rate analysis

DNA substitution rate comparative analysis was applied to ADS-related genes to obtain the genetic basis of the differential physiological responses of two congeneric species. A reciprocal best-hit method was adopted to support the orthologous relationship between each protein pair. Subsequently, local BLASTp (NCBI BLAST 2.2.26) was performed for further support, with an E-value threshold of 1.00 × 10^−5^ (Mu et al. 2015). Paired orthologous protein-coding sequences were aligned using ClustalW (https://www.ebi.ac.uk/Tools/msa/clustalo/). To examine the sequence divergence pattern of the orthologous proteins, we calculated nonsynonymous substitutions per nonsynonymous site (Ka), synonymous substitutions per synonymous site (Ks), and the ratio between Ka and Ks using the NG method implemented in KaKs_Calculator3.0 (Zhang 2022).

### Statistics analysis

To visualize the overall variations of ADS-related physiological indexes and gene expressions, we conducted Principal Component Analysis (PCA) using OriginPro 2022. The antioxidant indicators and changes in gene expression were presented as mean ± standard error (*n* = 3—5). Relative gene expression in treatment groups compared to the corresponding unstressed groups was normalized using the 2^−ΔΔcq^ method (Pfaffl 2001). For each gene, the transcriptional abundance of the unstressed group was standardized as 1.0. To assess the significance of pairwise comparisons between the treatment and unstressed groups, we conducted a *T*-test using SPSS (Version 25.0, SPSS Inc., United States) after normality testing and checking for homogeneity of variance through the Kolmogorov–Smirnov test.

## Results

### Overall redox balancing state under recurrent hypersaline stresses

**Fig. 2:**
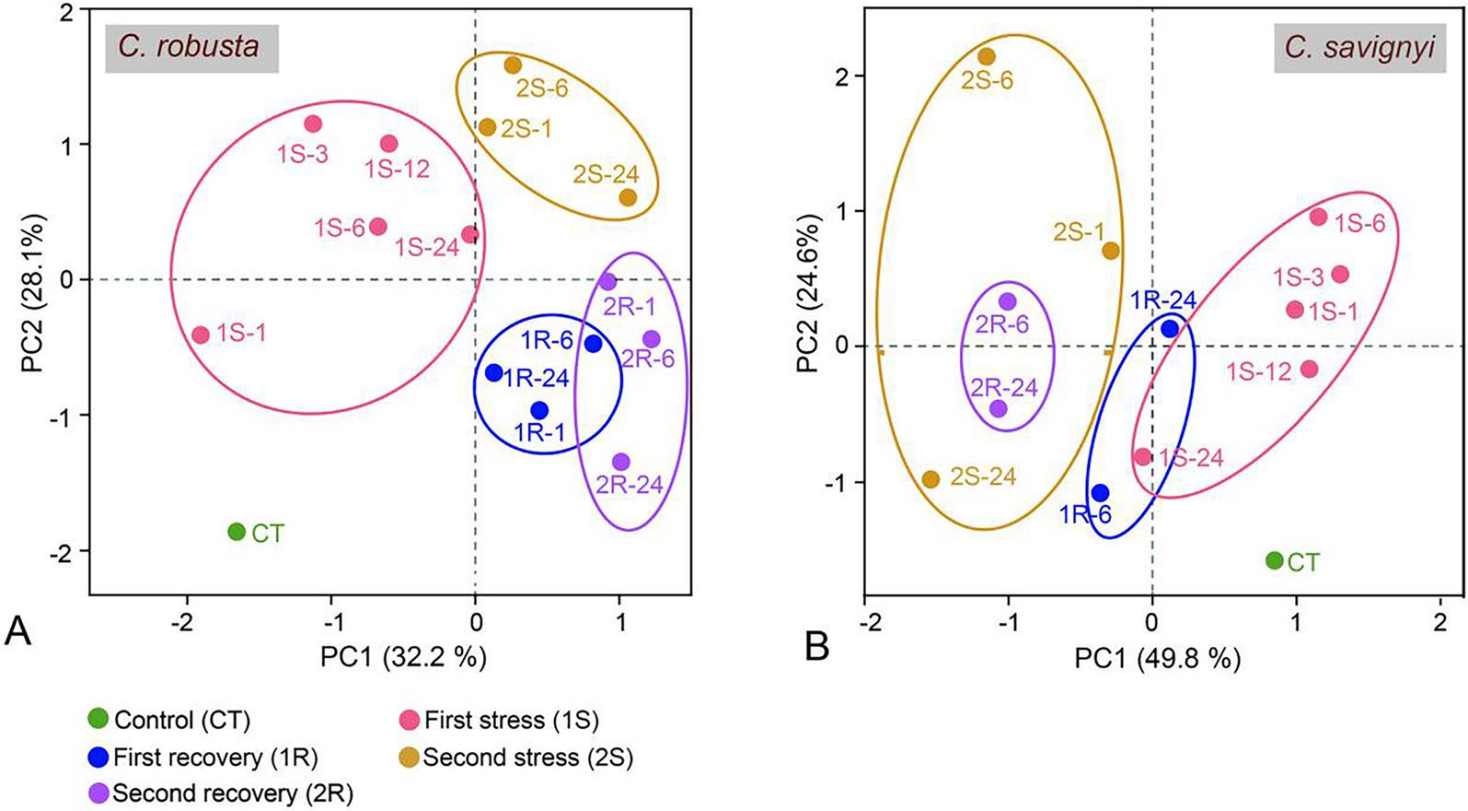
Principal component analysis (PCA) based on the results of antioxidant indicators of *C. robusta* (**A**) and *C. savignyi* (**B**). Each data point represents an experimental sample plotted using the first two principal components, with the coordinate axes indicating the first two principal component dimensions and their respective contributions to the overall variation among individuals. PCA was performed on the log-transformed, mean-centred data matrix

The PCA, which considered all indicators of oxidative stress and antioxidant capacity, revealed a rapid and distinct redox balancing process under two rounds of hypersaline stresses (Fig. 2). In *C. robusta*, the first two Principal Components (PCs) explained 32.2% and 28.1% of the total variation in the redox states of the ascidians. The individuals that underwent two cycles of hypersaline stresses (2S) were separated from those subjected to single hypersaline stress (1S, separated by axes PC1), as well as those in unstressed conditions (Fig. 2A). For *C. savignyi*, PC1 and PC2 explained 49.8% and 24.6% of the total variation, respectively. Similar to *C. robusta*, individuals of *C. savignyi* at the second stress-recovery stage were separated from those at the first stage (Fig. 2B). These results indicated that salinity changes induced oxidative stress and antioxidative responses, and previously experienced salinity changes had significant effects on the redox balancing process when ascidians encountered the same salinity stress again.

### Impacts of salinity stress on oxidative damage and antioxidant response

**Fig. 3:**
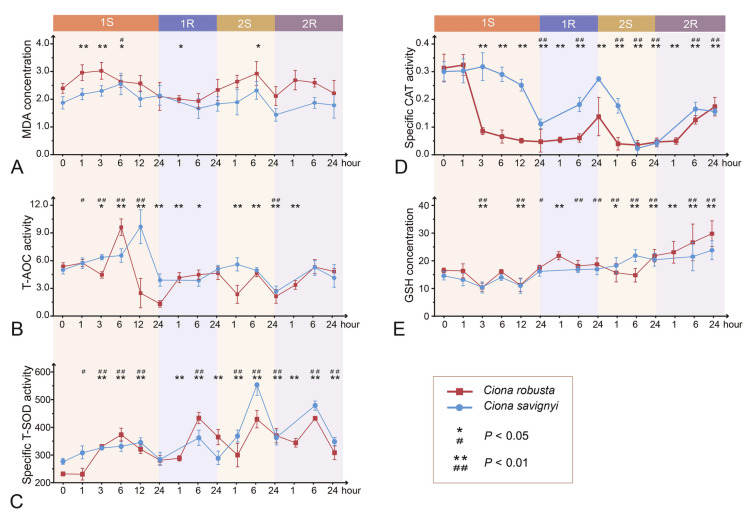
Effects of recurrent hyper-salinity challenges on oxidative damage (**A**), anti-oxidative level (**B**), and oxidative indicators (**C**–**E**). **A** MDA (malondialdehyde) concentration; **B** T-AOC (total antioxidative capacity) activity. **C** T-SOD (total superoxide dismutase) activity; **D** CAT (catalase) activity; **E** reduced GSH (glutathione) concentration; based on all samples from *C. savignyi* (blue line) and *C. robusta* (red line). Error bars represent the mean ± SE of six biological replicates. The significant differences between *C. robusta* and *C. savignyi* were denoted by asterisks (*) and pound signs (#): */#: *p* < 0.05, **/##: *p* < 0.01

Within each indicator, both *Ciona* species exhibited similar response patterns with significant positive correlations among all five indicators (*r* = 0.65–0.7 *p* < 0.05, Supplementary Table S2). *C. robusta* consistently exhibited higher MDA content than *C. savignyi* throughout the experiment, except at 1R-24 (Fig. 3A). In *C. robusta*, MDA content significantly increased at 1, 3, and 6 h after the first hypersaline stress and returned to the unstressed level afterward. During the second round of hypersaline stress, MDA content significantly increased at 6 h post-stress exposure and then returned to the unstressed level. However, MDA content in *C. savignyi* only showed a significant increase at 6 h during the first stress stage. In *C. robusta*, T-AOC content, a comprehensive indicator of antioxidative ability, increased 1.8-fold at 1S-6, then decreased to 1/2 and 1/6 of the control group at 1S-12 and 1S-24, respectively. T-AOC content eventually returned to the unstressed level at 1R-24. During the second stress, T-AOC fluctuated significantly but within a smaller range than during the first stress, and subsequently recovered to the unstressed level by the end of the experiment (Fig. 3B). Conversely, in *C. savignyi*, T-AOC showed a delayed significant increase at 1S-12 compared with *C. robusta* (1S-6) and then maintained at unstressed level during the first recovery period*.* The T-AOC changes during the second stress showed similar trend to that of *C. robusta* (Fig. 3B).

Under unstressed conditions, *C. robusta* had lower SOD activities compared to *C. savignyi* (Fig. 3C). After the first exposure to hypersaline stress, significantly increased SOD activity was observed in both *C. robusta* (at 3, 6, and 12 h) and *C. savignyi* (at 1, 3, 6, and 12 h). However, *C. robusta* exhibited a greater increase in SOD activity, surpassing that of *C. savignyi* at 6 h. Following the return to unstressed levels at 24 h after the first stress, SOD activity continued to rise during the first recovery stage. During the second stress-recovery stage, *C. savignyi* showed higher SOD activity at most stress treatment time points, with a greater rising amplitude compared to *C. robusta*. Furthermore, the rising amplitude of SOD activity in *C. savignyi* during the second stress stage was also greater than that observed during the first stress stage.

In contrast to the increase in SOD activity induced by stress, CAT activity significantly decreased at all time points after hypersaline treatments in both species, although there was a slight rebound at the two recovery stages (Fig. 3D). Notably, *C. robusta* consistently exhibited significantly lower CAT activities than *C. savignyi* at most experimental time points. The drop in CAT activity for *C. robusta* was more pronounced than that for *C. savignyi* during the first stress stage. After 1 h of exposure to high salinity, the CAT activity in *C. robusta* dropped to 1/6 of the control group and remained at a low level throughout the entire experiment. Conversely, *C. savignyi* experienced a sharp decrease to 1/3 of the control group by 12 h of treatment and returned to the unstressed level after the first 24-h recovery. These results suggest that the CAT activity should be strongly inhibited under hypersaline stress, and that *C. robusta*'s CAT activity should be more sensitive to salinity changes.

The overall GSH response patterns to recurrent hypersaline stresses in both species were similar. After the first round of hypersaline stress, GSH concentration significantly decreased at 3 and 12 h and then increased at 24 h. Starting from the first recovery stage, GSH concentration exhibited a steady increasing trend until the end of the whole experiment. For both species, the GSH concentration reached its maximum value at 2R-24, with levels twofold higher for *C. robusta* and 1.5-fold higher for *C. savignyi* compared to the control groups (Fig. 3E).

### Overall transcriptional changes of ADS-related genes under recurrent hypersaline stresses

**Fig. 4:**
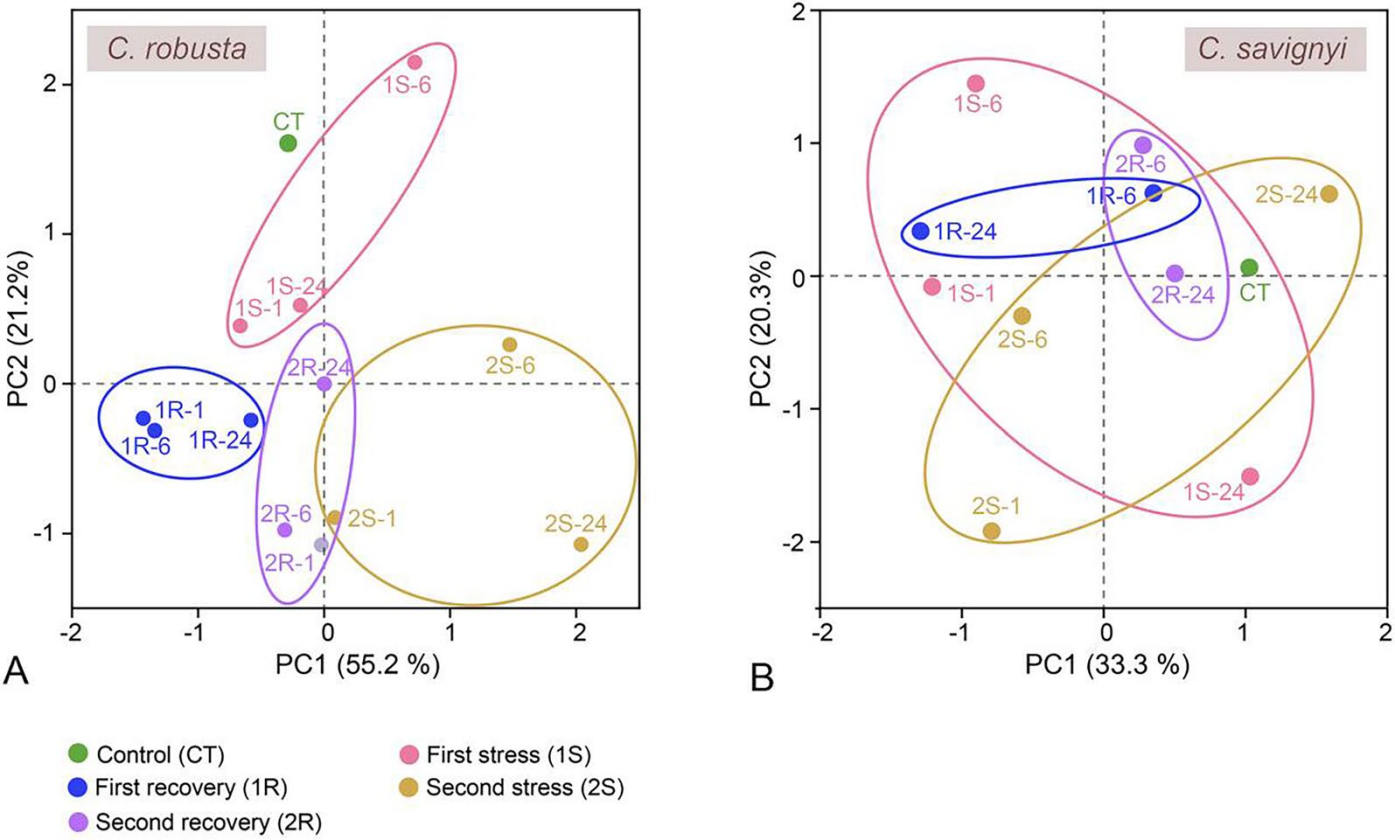
Principal component analysis (PCA) based on the results of gene expression of *C. robusta* (**A**) and *C. savignyi* (**B**). Each data point represents an experimental sample plotted using the first two principal components, with the coordinate axes indicating the first two principal component dimensions and their respective contributions to the overall variation among individuals. PCA was performed on the log-transformed, mean-centred data matrix

In *C. robusta*, the first two PCs explained 55.2% and 21.2% of the total variation in ADS-related gene expression. The samples from 1R & 2R and 1S & 2S were separated by axes PC1 and PC2, respectively. *C. robusta* samples were distinctly differentiated into two well-separated clusters, corresponding to the two rounds (1S1R & 2S2R) (Fig. 4A). Conversely, for *C. savignyi*, all groups were randomly scattered, and no clear stage- or treatment-dependent clusters were observed (Fig. 4B).

### Transcriptional response of the genes in the Nrf2-Keap1 signaling pathway

**Fig. 5:**
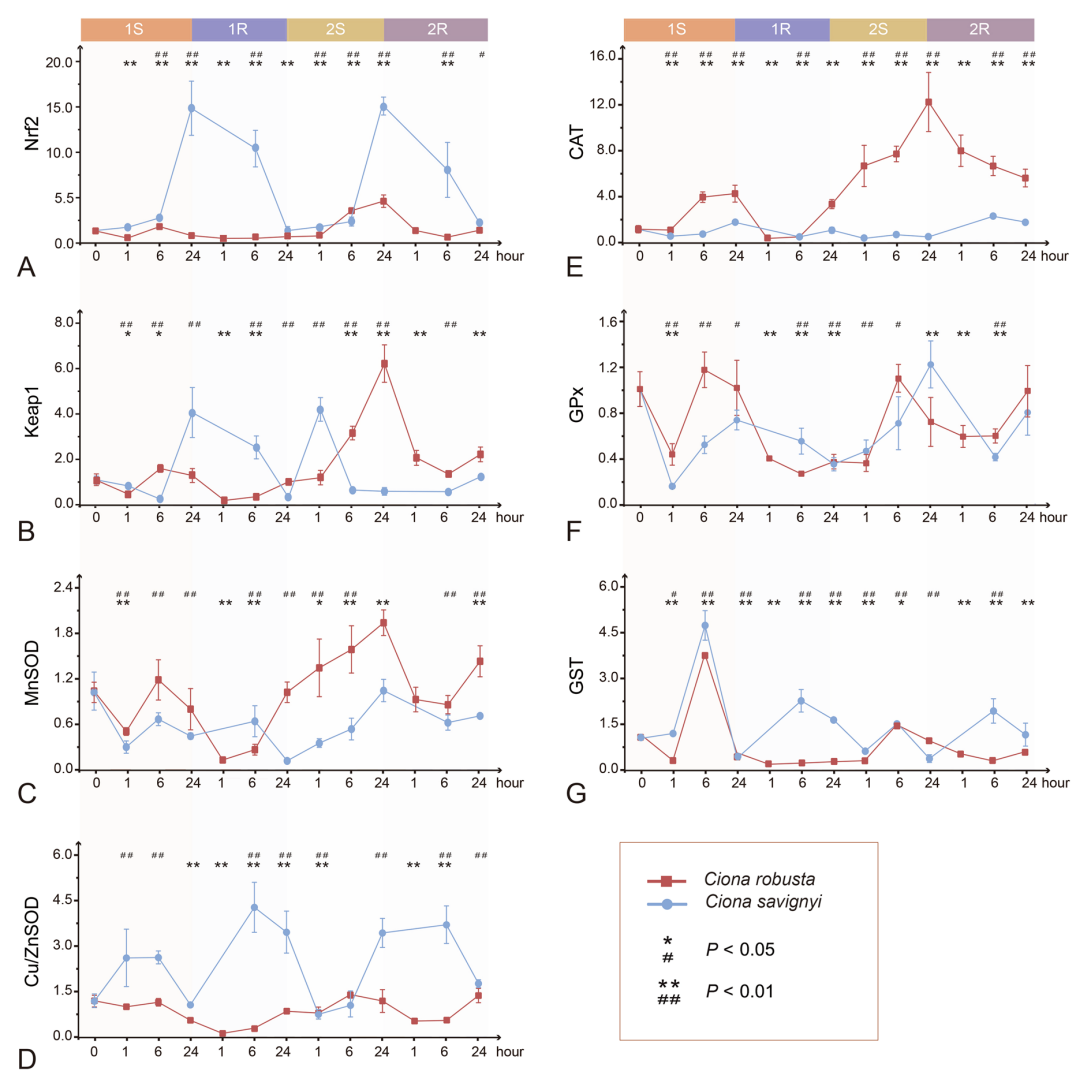
Effects of recurrent hypersalinity challenges on the relative expression level of Nrf2-keap1 signaling pathway**.** **A** Nuclear factor (erythroid-derived 2)-like 2 (Nrf2); **B** Kelch-like ECH-associated protein 1 (Keap1); **C** manganese superoxide dismutase (MnSOD); **D** copper/zinc superoxide dismutase (Cu/ZnSOD). **E** catalase (CAT); **F** glutathione S-transferases (GSTs); **G** glutathione peroxidase (GPx). Error bars represent the mean ± SE. The performance of *C. robusta* and *C. savignyi* were represented in red and blue lines, respectively. The significant differences between *C. robusta* and *C. savignyi* were denoted by asterisks (*) and pound signs (#): */#: *p* < 0.05, **/##: *p* < 0.01

In *C. robusta*, the expression levels of the transcriptional factors Nrf2 and Keap1 showed transient increases in both stress treatment groups (1S-6, 2S-6, and 2S-24, Fig. 5A, B). The amplitude of expression response during the repeated stresses was higher than that during the initial stress, indicating an enhanced response after the first round of stress. This pattern supports the formation of hypersalinity stress memory in *C. robusta*. In contrast, in *C. savignyi*, the expression of Nrf2 showed upregulations at all time points. Its highest expression levels during the first and repeated stress periods were both 14-fold higher than the control group at 1S-24 and 2S-24. Such levels were much higher than in *C. robusta* (Fig. 5A). Additionally, the expression of Keap1 showed significant upregulations at several time points, including 1S-24, 1R-6, and 2S-1. The peak value under the second stress (2S-1) appeared earlier than during the first stress (1S-24, Fig. 5B).

We further assessed the gene expression changes of five key ADS-related genes in the Nrf2-Keap1 signaling pathway (Fig. 5C–G). In *C. robusta*, the gene expression level of MnSOD significantly decreased at 1S-1, 1R-1, and 1R-6. It was continuously activated and showed a substantial increase during the second stress (Fig. 5C). However, the gene expression of Cu/ZnSOD was either unaffected or significantly decreased at most of the treatment groups (Fig. 5D). In *C. savignyi*, compared with the overall suppression state of MnSOD gene expression at most of the stress treatment groups, the gene expression level of Cu/ZnSOD was significantly induced at both stress-recovery stages, with a comparable rising range.

In *C. robusta*, CAT expression was significantly upregulated at both hypersaline stress stages. It then declined at the corresponding recovery stage, with a much higher increase during the second stress (Fig. 5E). In contrast, the CAT gene expression response of *C. savignyi* was relatively stable throughout the experiment, although some significant changes were detected at most stress treatment timepoints.

GPx and GST genes were selected for indicating GSH metabolism. GPx gene expression remained either unaffected or suppressed under hypersalinity stress-recovery treatments in both *C. robusta* and *C. savignyi* (Fig. 5F, G). GST gene expression was significantly induced at 6 h after the first and second hypersaline stresses in *C. robusta* and *C. savignyi*, with the transcriptional response amplitude lower under the second stress than that at the first stress (Fig. 5G). Notably, there was a difference in GST gene expression responses between the two species, with upregulation in *C. savignyi* and downregulation in *C. robusta* at the two recovery stages.

### Identification of orthologs and Ka/Ks ratios of ADS-related genes

Based on the results of reciprocal best hits, a total of 8222 putative orthologous pairs were identified, and all seven pairs of ADS-related genes were observed to have orthologous relationships. The average values of Ka, Ks, and Ka/Ks in the seven ADS-related gene pairs were 0.2444, 3.1690, and 0.0762, respectively (Supplementary Table S3). In comparison, across 8,222 orthologous gene pairs at the whole-genome level, the averages were calculated as 0.206 for Ka, 2.4168 for Ks, and 0.0709 for Ka/Ks. The pattern observed in the ADS-related genes indicates that they have experienced selective pressure favoring the conservation of protein-coding sequences. The lower Ka/Ks ratios suggest a prevalence of purifying selection, highlighting the functional importance and evolutionary stability of the ADS-related genes. However, Nrf2 exhibited a significantly elevated Ka/Ks ratio (Supplementary Table S3), suggesting a potential adaptive evolutionary outcome.

### Co-expression relationship between genes in the Nrf2-Keap1 signaling pathway

**Fig. 6:**
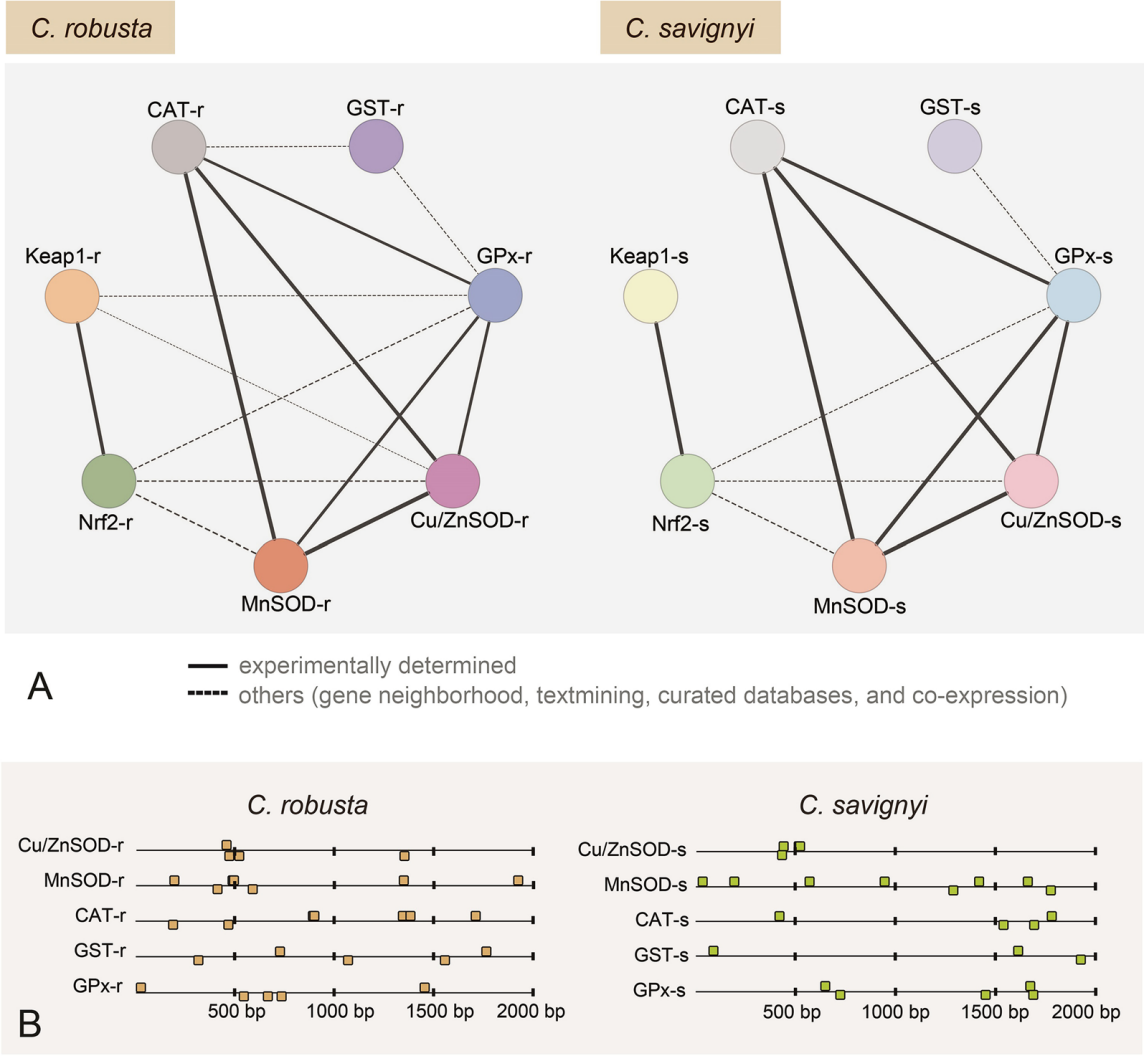
Comparative analysis of protein–protein interactions (PPI) and predicted antioxidant-responsive elements (AREs) between both *Ciona* species. **A** The PPI analysis of related proteins in the Nrf2-keap1 signaling pathways in *C. robusta* (left) and *C. savignyi* (right). Nodes represent proteins, edges represent direct and indirect interactions between proteins, and the width of the edges corresponds to the combined score. Experimentally determined interaction relationships are represented by solid lines, while interactions based on gene neighborhood, text mining, curated databases, and co-expression are indicated by dashed lines. B The numbers and distribution of identified AREs with the core sequence 5ʹ-G/ATGACNNNGC-3ʹ in downstream genes. AREs in *C. robusta* are represented by yellow cubes, while those in *C. savignyi* are depicted in green

Upon predicting the protein–protein interactions with genes on the Nrf2-Keap1 pathway, we observed a greater number of edges and average nodes in *C. robusta* (Fig. 6A; Supplementary Table S4). This pattern indicates the higher complexity of the Nrf2- keap1 pathway in *C. robusta*, which involves a higher number of protein–protein interactions. According to the results of antioxidant response element (ARE) prediction, the downstream genes in the pathway had 3–8 ARE sites within the 2000 bp upstream region of each sequence (Fig. 6B). We observed a higher number of ARE sites in the genes of *C. robusta* compared to those of *C. savignyi*, with an average of 5.2 and 4.6 within a 2000 bp region, and 3.2 and 2.2 within a 1000 bp region. Furthermore, our co-expression analysis revealed a significant relationship between the expression of Nrf2 and keap1 (*r* = 0.94, *p* < 0.01), as well as with downstream genes, including MnSOD, Cu/ZNSOD, and CAT (*r* > 0.6, *p* < 0.05, Supplementary Table S4). This demonstrates more intricate transcriptional regulations via Nrf2 and Nrf2-keap1 pathway in *C. robusta* than *C. savignyi,* and reveals a closer correlation between Nrf2 and its downstream genes and more finely tuned gene expression levels in *C. robusta*.

## Discussion

Given current global environmental changes and increasing human activities, it is beneficial to understand the mechanisms that govern rapid response to recurrent environmental stresses through multidimensional regulations. In this study, to understand the distinct antioxidative responses to environmental challenges in congeneric species, we investigated the impact of two rounds of 'hypersalinity exposure-recovery' experiments on two closely related ascidians, *C robusta* and *C. savignyi*, and further assessed antioxidative indicators and related gene expressions. Our results demonstrated that both *Ciona* species could rely on the activation of ADS by tuning antioxidant activity and/or gene expression in response to salinity fluctuations. *C. savignyi* exhibited higher tolerance and resistance to high salinity stresses at the physiological level, while *C. robusta* demonstrated advanced responses at the transcriptional level. Notably, both species exhibited stress memory in two dimensions. Specifically, the enhanced response of GSH and T-SOD was observed in both *Ciona* species after the first round of stress, indicating the physiological stress memory. The expressions of Nrf2, Keap1, MnSOD, and CAT displayed heightened responses, underlining the development of transcriptional stress memory. Furthermore, the two ascidians exhibited distinct preferences for SOD isoforms, with MnSOD significantly upregulated in *C. robusta* but Cu/ZnSOD in *C. savignyi*. This suggests differentiated regulatory mechanisms and defense strategies during salinity fluctuations in these two congeneric species. Collectively, the findings support the 'context-dependent stress memory hypothesis' that we proposed in this study, underscoring that the development of stress memory is species-specific at various regulation levels in response to recurring environmental challenges.

### Cellular damages and antioxidant scavengers under osmosis stresses

MDA concentration is an important indicator of oxidative damage, and its rapid accumulation has been extensively documented in response to various environmental stressors. For example, the MDA concentration significantly increased in *C. robusta* when salinity increased from 36 in spring to 40 in summer in Abu-kir Bay (Saad et al. 2011). Our results revealed the immediate MDA increase followed by recovery during the stress stages, indicating that hypersaline conditions transiently disrupted the balance between the excessive accumulation of ROS and detoxification. Furthermore, our results demonstrated the elevation of MDA with the activation of T-SOD at the same time points (Fig. 3A, C). This suggests that ROS may serve as a signaling factor in the activation of ADS to mitigate additional damages caused by excessive oxidation of lipid hydroperoxides (Chen et al. 2019; Zheng et al. 2016). Conversely, we observed a significant decrease in CAT activity (Fig. 3D, E), suggesting that CAT activity may be inhibited under high salinity conditions. This finding aligns with observations in *C. robusta* during seasonal hypersalinity fluctuations in the wild (Saad et al. 2011). Furthermore, we observed significant GSH accumulation throughout the entire experiment, particularly during the two recovery phases. Franchi et al. (2012) demonstrated continuous elevation of GSH synthase in *C. robusta* after 24 h of exposure to heavy metals such as Cu^2+^ and Cd^2+^, highlighting the significance of GSH biosynthesis as an efficient detoxification mechanism that enables *Ciona* to adapt and survive. These findings suggest that *Ciona* may prioritize its high capacity for GSH synthesis, which serves as an alternative process for ROS neutralization alongside CAT, particularly for scavenging H_2_O_2_ and lipid hydroperoxides in hypersaline environments.

Noticeably, in comparison to *C. savignyi*, we observed that MDA, T-AOC, and T-SOD in *C. robusta* reached the peaks earlier during the first stress (Fig. 3A–D), indicating a faster response than *C. savignyi*. Additionally, when compared with our previous study of *C. robusta* under recurrent cold stresses, we found that most antioxidative indicators induced by hypersalinity exposure reached resilience points earlier (after 6 h of exposure) than those induced by cold stresses (after 12 h of stress, Li et al. 2020). Several studies have demonstrated that faster and earlier responses can enhance population performance and survival probability (e.g., Wesener and Tietjen 2019). Given *C. robusta*'s higher baseline of MDA and lower baseline of T-SOD, the faster rate of regulation may imply that *C. robusta* is more sensitive to the initial salinity stress. Such a higher level of sensitivity allows this species to activate the ADS quickly, resulting in a robust physiological response. In contrast, *C. savignyi* exhibits higher tolerance and resistance to the initial stress, showing a more active ADS during the second stress phase.

### Differential transcriptional responses under osmosis stresses

The regulation of antioxidative-related genes, especially MnSOD and Cu/ZnSOD, represents a common response to oxidative stress induced by hypersalinity in marine species (Li et al. 2020; Zhang et al. 2014). Of note is the different transcriptional response patterns between *C. robusta* and *C. savignyi*. For example, *C. robusta* exhibited a preference for upregulating MnSOD, while *C. savignyi* favored inducing Cu/ZnSOD expression during salinity fluctuations (Fig. 5C, D). Cu/ZnSOD (also known as SOD1) and MnSOD (also known as SOD2) are typically found in the cytoplasm and mitochondria, respectively, and usually Cu/ZnSOD is the predominant SOD form which is post-translationally regulated by the copper chaperone (Kammer et al. 2011; Shadel and Horvath 2015). The upregulation of MnSOD over Cu/ZnSOD in *C. robusta* aligns with our observations in a previous study in *C. robusta* under cold stresses (Li et al. 2020), as well as with findings in mammals and fish species under abiotic stresses (Pietsch 2017; Röhrdanz and Kahl 1998). Collectively, available evidence suggests that *C. robusta* primarily relies on MnSOD to promptly catalyze the dismutation of ROS within the mitochondria generated by the electron transport chain. Moreover, Wang et al. (2016) conducted a comparison of the transcriptional profiles of two SOD isoforms in the eel *Anguilla marmorata* during 2-day hypersalinity challenges, and their findings revealed a significant upregulation of Cu/ZnSOD over MnSOD in the kidney. This alignment with our results in *C. savignyi* suggests that *C. savignyi* may employ alternative pathways within the cytoplasm to counter oxidative stresses, leading to substantial fluctuations in Cu/ZnSOD levels.

ADS responses encompass a range of multidimensional actions within a species, with physiological and transcriptional changes being two extensively studied regulatory layers. These layers may induce inconsistent responses, and their integrated effects can be considerably complex and context-dependent (Zhou and Wang 2023). For instance, we observed a reduction in CAT activity alongside an upregulation of CAT gene expression in both *Ciona* species (Fig. 3D, 5E). Hansen and colleagues reported a similar phenomenon in *Salmo trutta* after exposure to trace metals, where CAT activity was catalytically induced but not at the transcriptional level (Hansen et al. 2007). Several factors could contribute to this mismatch. Firstly, the transcription level of antioxidant enzymes may reflect instantaneous activity, while there could be a time-lag effect between mRNA expression and enzyme protein synthesis throughout the experiment (Chen et al. 2019; Zhang et al. 2017). Secondly, the presence of isoenzymes with multi-copy genes can lead to differential alterations between these two levels (Han et al. 2013). Finally, the enzyme activity can be influenced by various regulatory factors, including post-transcriptional and post-translational modifications (Cao et al. 2003).

The species-specific responses to recurrent environmental challenges observed at both levels raise several questions, such as those associated with the drivers and associated mechanisms responsible for the divergent responses. Clearly, answering these questions based on findings from this study and available literature is not straightforward, as few studies have directly compared the comprehensive responses to environmental stresses at different regulation levels among different species in the same system. So far, the available evidence remains independent and fragmented. These two *Ciona* species were estimated to have diverged around 120 million years ago (Delsuc et al. 2018). They exhibit some distinct morphological features, including the two-fold stiffness of the tunic and a greater number of tentacles around the buccal siphon in *C. robusta* compared to *C. savignyi* (Hoshino and Nishikawa 1985; Tarallo et al. 2016). Despite the potential influence of these features on their performance under osmotic stresses, there is currently no direct evidence bridging these features with their divergent osmotic responses. Therefore, future investigations could evaluate the causes and consequences of context-dependent stress memory observed in this study, particularly from a comprehensive perspective at multiple regulation layers.

### Stress memory based on antioxidant defense system

Stress memory refers to the mechanisms developed by organisms to recall previous instances of stresses and adjust their responses, leading to more efficient reactions when faced with repeated stresses (Avramova 2015; Crisp et al. 2016; Hackerott et al. 2021; Hilker and Schmulling 2019). Such a memory is particularly valuable for sessile organisms that passively experience complex environments, as it represents an effective strategy to resist recurrent environmental stresses (Hackerott et al. 2021; Hilker and Schmulling 2019). Our findings in this study emphasize that the formation of stress memory is influenced by both the species and environmental challenges they face, supporting the 'context-dependent stress memory hypothesis'.

At the transcriptional level, a comparison of gene expression responses in ADS-related genes between the two rounds of stress revealed that *C. robusta* exhibited more pronounced transcriptional responses in the Nrf2-Keap1 pathway (including Nrf2, Keap1, MnSOD, and CAT) during the second hypersalinity stress; however, this was not observed in *C. savignyi* (Fig. 5A–E; Supplementary Table S4). These findings underscore that *C. robusta* developed transcriptional stress memory from the earlier stress, supporting the notion of species-specific transcriptional stress memory. A similar phenomenon was reported in *C. robusta*: pre-exposure to Lipopolysaccharide (LPS) enhanced the transcriptional response of antioxidative-related genes in the gut (e.g., Cu/ZnSOD and GST) during later hypoxia/starvation (H/S) stresses (Marino et al. 2023). The regulation of the ADS-related signaling pathway is critical for increasing the antioxidant capacity and membrane stability, which, in turn, reduces levels of malonaldehyde and the production of superoxide radicals. This provides a higher level of protection for maintaining stress memory in response to various environmental stressors (Hilker and Schmulling 2019; Wang et al. 2018, 2019).

Several studies have provided insight into the molecular mechanisms that underlie transcriptional stress memory, such as sustained modifications of key transcriptional factors that mediate cascading effects on targeted genes within the same signaling pathway (Liu et al. 2022; Mozgova et al. 2019). When combining the findings of this study with our previous work in *C. robusta*, we identified that the transcription factors Nrf2 and Keap1 within the Nrf2-Keap1 pathway exhibited stress memory profiles. These profiles were positively correlated with the expression patterns of downstream genes (Fig. 5; Supplementary Table S4, Li et al. 2020). As a result, the emergence of stress memory within the Nrf2-Keap1 pathway in *C. robusta* is closely associated with Nrf2 and Keap1. The enhanced upregulation of Nrf2 could act as the potential 'memory factor', further mediating the increased stress memory within downstream genes during the second stress phase. Conversely, in the context of the 'non-memory' profile, there were consistent expression levels of transcription factors in *C. savignyi* during both rounds of stresses (Fig. 5). However, considering the limited number of selected genes in the study, further investigation involving the systematic screening of the whole transcriptome may provide a better understanding of the mechanism of transcriptional stress memory between the two *Ciona* species. Also, a more extensive screening would enable the identification of additional genes and pathways involved in stress memory, potentially offering deeper insights into the underlying mechanisms governing the response to environmental challenges. Our predictions from the PPI and ARE analysis also illustrated regulatory distinctions within the Nrf2-Keap1 pathway between these two *Ciona* species (Fig. 6; Supplementary Table S5). These differences might be attributed to the evolutionary history of Nrf2 in *C. robusta*. In the test of Ka/Ks, the ratio of the transcription factor Nrf2 was higher compared to the rest of the ADS-related genes (0.17 vs. 0.07, Supplementary Table S3). This pattern suggests that the selective pressure on Nrf2 may result from its pivotal role in mediating adaptive responses to environmental challenges, underscoring the significance of its function in maintaining cellular homeostasis. The selective pressures and environmental conditions encountered during the evolution of Nrf2 have resulted in an increased number of ARE sites and an expansion or addition of more interactions within the Nrf2-Keap1 pathway in *C. robusta*. The disparity of the ADS between both *Ciona* species offers a possible explanation for the lack of transcriptional stress memory in *C. savignyi* during recurrent stress events.

In comparison to our prior study in *C. robusta* under cold stresses, we found that both recurrent hypersalinity and cold stresses could induce transcriptional stress memory through the ADS, albeit via distinct trajectories (Li et al. 2020). Specifically, in *C. robusta*, the expression of ADS-related genes notably decreased during subsequent cold stresses, while it increased upon the second exposure to hypersalinity compared to the initial salinity stress. This suggests that stress memory patterns can be specific to the type of stress, further supporting the 'context-dependent stress memory hypothesis'. Both salinity and temperature are recognized as primary environmental factors influencing the distribution of *C. robusta* (Carver et al. 2006; Paiva et al. 2018; Renborg et al. 2013). The trajectory of transcriptional stress memory reflects the changing direction of expression levels following integrated adaptive processes in response to distinct environmental cues, which are intricately linked to the molecular mechanisms that underlie the induction of memory. Furthermore, their substantial extent suggests comprehensive consequences in the short term and overall effects at the organismal level. In vertebrates, the initial stress can prime the ADS, making it more responsive to subsequent challenges, a phenomenon known as potentiation, or less intense than the first one, referred to as tolerance (Melillo et al. 2018). It is worth emphasizing that stress memory implies an evolutionary trajectory and provides potential evidence for evaluating an organism's adaptive dynamics (Avramova 2015; Brown and Barott 2022).

In addition, stress memory is defined as a multidimensional phenomenon, encompassing multiple layers of processes in the response. At the physiological level, *C. robusta* exhibited an increased baseline level of SOD activity across four stages, while *C. savignyi* indicated an enhanced response during the second round of stress (Fig. 3C). Both *Ciona* species demonstrated a continuous increase in GSH concentration throughout the entire experiment (Fig. 3E). These findings unveil diverse patterns involving multiple indicators, considered as the physiological stress memory of the ADS in *Ciona* species. Following recurrent exposures to UV, arthropods such as *Daphnia magna* adapted strategies related to the plastic upregulation of key antioxidant enzymes. These strategies encompass the development of a higher baseline in GST, an increase in plasticity in GPx, a decrease in plasticity in CAT, and the loss of plasticity in SOD (Oexle et al. 2016). Hence, it is beneficial to use a multidimensional and multivariate approach when investigating intricate defense systems such as the ADS. This approach not only enhances our comprehensive understanding of the adaptive responses of the ADS to environmental perturbations but also illuminates the mechanisms that underlie the rapid adaptation of the ADS to fluctuating environmental conditions.

The capacity to acquire evolved plasticity in response to stressful environments is important in predicting population dynamics and distribution patterns (Snell-Rood et al. 2018; Xue and Leibler 2018; Zhou and Wang 2023). For example, research on recurring coral bleaching, particularly within the *Acropora* genus, has shown that transcriptional resilience is instrumental in sensitivity and contributes to potential bleaching stressors. *Acropora gemmifera*, which exhibits significantly higher fitness and recovery rates compared to *A. hyacinthus*, displayed greater resistance during repeated heat extremes (Thomas et al. 2019). From a biological invasion perspective, stress memory equips living organisms with high levels of stimulus-induced plasticity, especially through the ADS derived from prior experiences. This allows invaders to modulate and refine their performance during the invasion process. A previous study on the highly invasive angiosperm *Solidago canadensis* has revealed a close relationship between antioxidative ability through pre-adaption and invasiveness (Cheng et al. 2021). Thus, in the face of unpredictable environmental challenges, species that benefit from enhanced performance due to stress memory may demonstrate greater adaptability, resulting in higher fitness and increased survival potential.

## Conclusions

Our comparative assessment indicates that marine invasive species employ the synergistic regulation of the multidimensional network within the ADS to maintain homeostasis. Our results reveal that hypersalinity stresses induced a similar physiological response pattern in both species. However, the species exhibited varying responses to identical environmental challenges—*C. robusta* displayed higher sensitivity during the initial stress while *C. savignyi* exhibited greater tolerance. Each species employed different transcriptional strategies to combat oxidative stress during recurrent hypersalinity challenges, as evident in their preferences for SOD isoforms. Physiological stress memory was observed in both ascidians and *C. robusta* exhibited an amplified response to recurring stresses. All the findings presented here, in conjunction with our previous results, support the 'context-dependent stress memory hypothesis' that we proposed in this study. This hypothesis demonstrates that when species cope with recurrent environmental challenges, the formation of stress memory is species-specific and its manifestation is environmental stress-dependent.

### Supplementary Information

Below is the link to the electronic supplementary material.

Supplementary file1 (DOCX 35 KB)

## Data Availability

The data supporting this study are available from the corresponding author upon reasonable request.
