# Supplementary material for: Context-dependent antioxidant defense system (ADS)-based stress memory in response to recurrent environmental challenges in congeneric invasive species
Source: Mar Life Sci Technol. 2024 May 8;6(2):315–30. doi: 10.1007/s42995-024-00228-y (PMC11136907; doi:10.1007/s42995-024-00228-y)
Supplement: Supplementary file 1 — Supplementary file1 (DOCX 35 KB) [file 42995_2024_228_MOESM1_ESM.docx]

**Supplementary Information**

**Journal: Marine Life Science & Technology**

**Article title: Context-dependent antioxidant defense system (ADS)-based stress memory in response to recurrent environmental challenges in congeneric invasive species**

**Hanxi Li^1,2^, Xuena Huang^1^, Aibin Zhan ^1,2^**^*^

^1^ Research Center for Eco-Environmental Sciences, Chinese Academy of Sciences, 18 Shuangqing Road, Haidian District, Beijing 100085, China

^2^ University of Chinese Academy of Sciences, 19A Yuquan Road, Shijingshan District, Beijing 100049, China

* Correspondence: Prof. Aibin Zhan; Email: zhanaibin@hotmail.com or azhan@rcees.ac.cn; Phone & Fax: (+86)-10-6284-9882.

**Supplementary Table S1.** Primer sequences for the genes used in the real-time PCR analysis

|  | Gene ID | Forward primer（5‘-3’） | Reverse primer（5‘-3’） | PCR efficiency |
| --- | --- | --- | --- | --- |
| β-actinR | ENSCING00000008797 | ACCGGCTTCAGCTGCTAAGTAAC | TGTCCGATGTATTGGTCCTTTATTG | 1.93 |
| MnSODR | ENSCING00000004261 | GTTGGGTCTGGATAAAGAAGCC | TTTGGACGGAAGTTCTTGTATTGT | 2.00 |
| Cu/ZnSODR | ENSCING00000021568 | GACGGCAGATTCAAGTGGTGTAG | TGTTGTCTTGCTTTGTGGATGAG | 1.98 |
| CATR | ENSCING00000005187 | TCGTGGGTTCGCCATCAAGT | TAAGATGAGTTTGCGGGTTTCG | 1.92 |
| GSTR | ENSCING00000003309 | TCTTTATGTTTCGCTTGCTTGTC | GATCTTTCTCGCATTGCTCAAC | 1.91 |
| GPxR | ENSCING00000012924 | ATCGGAAAGGTTTCACTCGTTG | CTGATTACACGGGAAAGCAAGG | 2.00 |
| Nrf2R | ENSCING00000003373 | AACTCGGACACTGACGAAGCAT | TTTATGGAGAACGGAAGGACAAC | 1.97 |
| Keap1R | ENSCING00000008356 | CTTGGTCTTGCTGCGTTTGC | CTTCCGATTGCGACACCTGTT | 1.94 |
| RPL17S | ENSCSAVT00000017678 | CGGAGTTGGTCGTTGTGCC | CCTTGACATCGGCGTTGCT | 1.82 |
| MnSODS | ENSCSAVG00000011007 | CACCTTACCAGATTTGCCATACG | TTGCCTCAGCTTCATGCAGTTT | 1.98 |
| Cu/ZnSODS | ENSCSAVG00000003596 | CCGACAACCTTGTGACGCTAAC | ATGACGCCACATGCCAATCTAC | 1.87 |
| CATS | ENSCSAVG00000001204 | CATACTTTCAAAATGGTCAACGC | TCACGTATTGAGTGGTCTGGGT | 2.01 |
| GSTS | ENSCSAVG00000008704 | CTGTATGTTTCCCTTGCTTGTCC | CTCCAGCCATTGTCAAGCATC | 1.93 |
| GPxS | ENSCSAVG00000004929 | TTGAACAATATGCCGGAAAGAT | GGGAACGCAAGGACAGTGAAT | 2.00 |
| Nrf2S | ENSCSAVG00000003043 | TTCCAGACAGACAGACCCAGTTC | TGGTTGATGTTCGGTTGTAGTTG | 1.99 |
| Keap1S | ENSCSAVG00000011359 | GGATGAGGTGCGTGTGGATT | ACCGCTTCCAGCAGCATCTT | 1.98 |

*β*-actinR and RPL17S are the housekeeping genes for the calculation of relative expression levels. Homologous genes with the suffix ‘R’ and ‘S’ presented the genes that belonged to *C. robusta* and *C. savignyi*, respectively.

**Supplementary Table S2.** The correlation coefficients (above diagonal) and the *P* values (below diagonal) of physiological indexes of both *Ciona* species. *: *p* < 0.05, **: *p* < 0.01. -r = *Ciona robusta*, -s = *Ciona* *savignyi*

|  | SOD-r | SOD-s | CAT-r | CAT-s | GSH-r | GSH-s | MDA-r | MDA-s | AOC-r | AOC-s |
| --- | --- | --- | --- | --- | --- | --- | --- | --- | --- | --- |
| SOD-r | ---- | 0.694^*^ | -0.666^*^ | -0.463 | 0.181 | 0.484 | -0.107 | -0.086 | 0.129 | -0.136 |
| SOD-s | 0.018 | ---- | -0.424 | -0.627^*^ | 0.125 | 0.656^*^ | 0.413 | 0.119 | 0.02 | 0.023 |
| CAT-r | 0.025 | 0.194 | ---- | 0.672^*^ | 0.061 | -0.387 | 0.263 | 0.005 | 0.31 | 0.27 |
| CAT-s | 0.152 | 0.039 | 0.024 | ---- | -0.201 | -0.725^*^ | 0.187 | 0.251 | 0.606^*^ | 0.649^*^ |
| GSH-r | 0.594 | 0.714 | 0.858 | 0.553 | ---- | 0.683^*^ | -0.348 | -0.489 | -0.062 | -0.36 |
| GSH-s | 0.131 | 0.028 | 0.24 | 0.012 | 0.021 | ---- | -0.12 | -0.379 | -0.314 | -0.427 |
| MDA-r | 0.753 | 0.207 | 0.434 | 0.582 | 0.295 | 0.726 | ---- | 0.651^*^ | 0.418 | 0.740^**^ |
| MDA-s | 0.802 | 0.727 | 0.988 | 0.457 | 0.127 | 0.251 | 0.03 | ---- | 0.564 | 0.734^*^ |
| AOC-r | 0.706 | 0.954 | 0.353 | 0.048 | 0.857 | 0.347 | 0.201 | 0.071 | ---- | 0.700^*^ |
| AOC-s | 0.69 | 0.946 | 0.422 | 0.031 | 0.276 | 0.19 | 0.009 | 0.01 | 0.017 | ---- |

**Supplementary Table S3.** Orthologous relationships and Ka, Ka, Ka/Ks of *Ciona robusta* and *C. savignyi*.

| Gene name | GeneTree_ID | Type | Target %id | Query %id | GOC Score | WGA Coverage | High Confidence | Ka | Ks | Ka/Ks | *P*-Value  (Fisher) |
| --- | --- | --- | --- | --- | --- | --- | --- | --- | --- | --- | --- |
| MnSOD | ENSGT00390000011877 | 1-to-1 | 61.75 % | 77.01 % | 50 | 100 | Yes | 0.1331 | 2.1122 | 0.0630 | 1.09E-36 |
| Cu/ZnSOD | ENSGT00940000168521 | 1-to-1 | 71.79 % | 71.79 % | 75 | 99.31 | Yes | 0.2187 | 2.0029 | 0.1092 | 9.43E-25 |
| CAT | ENSGT00390000018100 | 1-to-1 | 80.81 % | 82.23 % | 100 | 92.98 | Yes | 0.1239 | 2.0520 | 0.0604 | 5.11E-35 |
| GST | ENSGT00530000065151 | 1-to-1 | 75.63% | 76.06% | 100 | 100 | Yes | 0.2076 | 2.7802 | 0.0747 | 3.33E-32 |
| GPx | ENSGT00940000174213 | 1-to-1 | 71.58% | 71.96% | 75 | 90.09 | Yes | 0.1487 | 4.0712 | 0.0365 | 1.18E-86 |
| Nrf2 | ENSGT00950000182892 | 1-to-1 | 51.84 % | 51.96 % | 50 | 73.93 | Yes | 0.8107 | 4.6427 | 0.1746 | 7.51E-40 |
| Keap1 | ENSGT00940000170765 | 1-to-1 | 88.79% | 89.10% | 98.71 | 98.71 | Yes | 0.0682 | 4.5220 | 0.0151 | 3.69E-214 |

The *p*-value (Fisher) was computed using the Fisher exact test based on synonymous and nonsynonymous sites (S-Sites and N-Sites) and synonymous and nonsynonymous substitutions (S-Substitutions and N-Substitutions).

**Supplementary Table S4.** The correlation coefficients (above diagonal) and the *P* values (below diagonal) of genes related to the Nrf2-Keap1 pathway in both *Ciona* species *: *p* < 0.05, **: *p* < 0.01. -r = *Ciona robusta*, -s = *Ciona* *savignyi.*

|  | MnSOD-r | MnSOD-s | Cu/ZnSOD-r | Cu/ZnSOD-s | CAT-r | CAT-s | GPx-r | GPx-s | GST-r | GST-s | Nrf2-r | Nrf2-s | Keap1-r | Keap1-s |
| --- | --- | --- | --- | --- | --- | --- | --- | --- | --- | --- | --- | --- | --- | --- |
| MnSOD-r | ---- | 0.38 | 0.721* | -0.332 | 0.848** | -0.117 | 0.444 | 0.615* | 0.304 | -0.202 | 0.812** | -0.015 | 0.840** | -0.229 |
| MnSOD-s | 0.248 | ---- | 0.329 | 0.034 | 0.299 | 0.044 | 0.451 | 0.834** | 0.303 | -0.02 | 0.548 | 0.325 | 0.545 | -0.222 |
| Cu/ZnSOD-r | 0.012 | 0.324 | ---- | -0.43 | 0.345 | -0.158 | 0.612* | 0.405 | 0.455 | -0.002 | 0.621* | -0.46 | 0.51 | -0.515 |
| Cu/ZnSOD-s | 0.319 | 0.922 | 0.187 | ---- | -0.066 | -0.001 | -0.513 | -0.206 | -0.122 | 0.35 | -0.064 | 0.316 | 0.027 | -0.439 |
| CAT-r | 0.001 | 0.373 | 0.299 | 0.847 | ---- | 0.007 | 0.2 | 0.491 | 0.069 | -0.29 | 0.762** | 0.36 | 0.881** | -0.104 |
| CAT-s | 0.733 | 0.898 | 0.643 | 0.998 | 0.983 | ---- | 0.291 | 0.015 | -0.186 | -0.073 | -0.309 | 0.153 | -0.158 | -0.053 |
| GPx-r | 0.171 | 0.164 | 0.045 | 0.107 | 0.555 | 0.385 | ---- | 0.502 | 0.682* | 0.208 | 0.438 | -0.024 | 0.314 | -0.213 |
| GPx-s | 0.044 | 0.001 | 0.217 | 0.544 | 0.125 | 0.964 | 0.116 | ---- | 0.16 | -0.344 | 0.695* | 0.43 | 0.721* | -0.015 |
| GST-r | 0.363 | 0.365 | 0.160 | 0.721 | 0.840 | 0.584 | 0.021 | 0.639 | ---- | 0.743** | 0.366 | -0.178 | 0.187 | -0.376 |
| GST-s | 0.552 | 0.954 | 0.996 | 0.291 | 0.388 | 0.831 | 0.539 | 0.301 | 0.009 | ---- | -0.114 | -0.278 | -0.263 | -0.429 |
| Nrf2-r | 0.002 | 0.081 | 0.041 | 0.852 | 0.006 | 0.355 | 0.178 | 0.018 | 0.268 | 0.739 | ---- | 0.267 | 0.940** | -0.37 |
| Nrf2-s | 0.965 | 0.329 | 0.155 | 0.344 | 0.277 | 0.652 | 0.944 | 0.187 | 0.601 | 0.408 | 0.427 | ---- | 0.417 | 0.302 |
| Keap1-r | 0.001 | 0.083 | 0.109 | 0.937 | 0.000 | 0.642 | 0.346 | 0.012 | 0.581 | 0.435 | 0.000 | 0.202 | ---- | -0.289 |
| Keap1-s | 0.497 | 0.511 | 0.105 | 0.177 | 0.761 | 0.876 | 0.529 | 0.965 | 0.255 | 0.188 | 0.263 | 0.366 | 0.388 | ---- |

**Supplementary Table S5.** Confidence scores between the targeted indicators calculated by STRING v.12. The numbers above and below the diagonal represent the confidence score for *Ciona robusta* and *C. savignyi*, respectively.

| *C. robusta*  *C. savignyi* | Cu/ZnSOD | MnSOD | CAT | GPx | GST | Nrf2 | Keap1 |
| --- | --- | --- | --- | --- | --- | --- | --- |
| Cu/ZnSOD | ---- | 0.992 | 0.863 | 0.68 | / | 0.315 | 0.165 |
| MnSOD | 0.99 | ---- | 0.921 | 0.697 | / | 0.366 | / |
| CA | 0.934 | 0.955 | ---- | 0.63 | 0.219 | / | / |
| GPx | 0.824 | 0.891 | 0.798 | ---- | 0.24 | 0.304 | 0.216 |
| GST | / | / | / | / | ---- | / | / |
| Nrf2 | 0.0.26 | 0.315 | / | 0.25 | / | ---- | 0.823 |
| Keap1 | / | / | / | / | / | 0.885 | ---- |
